# Human resources for maternal, newborn and child health: from measurement and planning to performance for improved health outcomes

**DOI:** 10.1186/1478-4491-9-16

**Published:** 2011-06-24

**Authors:** Neeru Gupta, Blerta Maliqi, Adson França, Frank Nyonator, Muhammad A Pate, David Sanders, Hedia Belhadj, Bernadette Daelmans

**Affiliations:** 1 Health Workforce Information and Governance, World Health Organization, Geneva, Switzerland; 2 Making Pregnancy Safer, World Health Organization, Geneva, Switzerland; 3 Ministry of Health of Brazil, Brasilia, Brazil; 4 Policy, Planning, Monitoring and Evaluation Division, Ghana Health Service, Accra, Ghana; 5 National Primary Health Care Development Agency, Abuja, Nigeria; 6 School of Public Health, University of the Western Cape, Cape Town, South Africa; 7 Partnerships Department, UNAIDS, Geneva, Switzerland; 8 Newborn and Child Health and Development, World Health Organization, Geneva, Switzerland

## Abstract

**Background:**

There is increasing attention, globally and in countries, to monitoring and addressing the health systems and human resources inputs, processes and outputs that impede or facilitate progress towards achieving the Millennium Development Goals for maternal and child health. We reviewed the situation of human resources for health (HRH) in 68 low- and middle-income countries that together account for over 95% of all maternal and child deaths.

**Methods:**

We collected and analysed cross-nationally comparable data on HRH availability, distribution, roles and functions from new and existing sources, and information from country reviews of HRH interventions that are associated with positive impacts on health services delivery and population health outcomes.

**Results:**

Findings from 68 countries demonstrate availability of doctors, nurses and midwives is positively correlated with coverage of skilled birth attendance. Most (78%) of the target countries face acute shortages of highly skilled health personnel, and large variations persist within and across countries in workforce distribution, skills mix and skills utilization. Too few countries appropriately plan for, authorize and support nurses, midwives and community health workers to deliver essential maternal, newborn and child health-care interventions that could save lives.

**Conclusions:**

Despite certain limitations of the data and findings, we identify some key areas where governments, international partners and other stakeholders can target efforts to ensure a sufficient, equitably distributed and efficiently utilized health workforce to achieve MDGs 4 and 5.

## Background

In June 2010, leaders of the G8 nations announced a comprehensive and integrated approach to accelerate progress towards the Millennium Development Goals (MDGs) 4 and 5 for maternal and child health (known as the Muskoka Declaration) [1]. The initiative aimed to support strengthening of national health systems in developing countries, in order to enable accelerated delivery of key interventions for improved maternal, newborn and child health (MNCH) outcomes along the continuum of care. The Global Strategy for Women's and Children's Health, launched at the United Nations MDG Summit on 22 September 2010, provided a significant opportunity to broaden these commitments [2]. With only four years left until the 2015 deadline to achieve the MDGs, this year presents a critical opportunity for action to increase investment and support to countries to strengthen their basic health systems, including their health workforce, to deliver essential health services that could save the lives of women and children.

There is an accumulating body of evidence that increased availability of skilled health workers is directly linked to improved MNCH outcomes [3-5]. However there is tremendous variation across countries not only in availability and distribution of doctors, nurses, midwives and other trained providers, but also of the services actually provided by health workers with the same occupational title. This paper focuses on an area critical to policymakers, implementers and donors, namely the collection and use of strategic information on human resources for health (HRH) for decision making and performance monitoring to achieve the MDGs for maternal and child health.

Improved reporting and validation processes are necessary to ensure that progress is achieved and sustained and that all partners are meeting their commitments. We collate and analyse new and existing quantitative and qualitative data on the availability, distribution, roles and functions of human resources in 68 low- and middle-income countries that together account for over 95% of maternal and child deaths worldwide. Special attention is given to the HRH factors that can accelerate or hinder progress to reach MDGs 4 and 5. We also review innovative strategies and lessons learnt from countries that have used data and information to appropriately plan for and monitor HRH performance to accelerate action to improve MNCH outcomes.

## Framework and methods

The paper builds on work of the Countdown to 2015 Initiative, a global independent collaboration of concerned individuals and partner organizations that tracks progress made towards the achievement of MDGs 4 and 5, and promotes the use of evidence to enhance decision and policy making and increase health investments at the country level [6,7]. In 2008, the Countdown identified 68 priority countries in different regions of the world for action on maternal, newborn and child health [6]. We focus on health workforce development as a critical factor in the effective delivery of the continuum of care for MNCH among these 68 countries.

In line with existing efforts by many countries in monitoring their progress, the Countdown tracks a series of indicators of coverage of key interventions proven effective in reducing maternal, newborn and child mortality, as well as indicators of health systems and policies, financial flows and equity [6-8]. Among the indicators of health systems and policies, two core indicators related to HRH for MNCH have been identified and are being regularly monitored [9]. The first is density of doctors, nurses and midwives in the country; the second, existence of a policy or guideline authorizing midwives to perform a set of signal functions for basic emergency obstetric and neonatal care. This study reviews and synthesizes the latest available data on these two indicators, and presents further analyses with complementary information from national and international sources.

The data source of the workforce density indicator is the World Health Organization's Global Atlas of the Health Workforce [10]. This database collates HRH statistics from official national sources, including administrative records, population censuses and other statistical surveys. Workforce density provides information on the stock of health workers relative to the population, and can be used to assess whether it meets a minimum threshold necessary to provide basic health care coverage. We present fresh data on density of doctors, nurses and midwives across the Countdown priority countries, and a new analysis on geographical distribution within countries. Our findings refer only to three occupation groups, those for which data are most complete and comparable internationally. Geographical distribution of HRH is measured by rural/urban, and weighted by population figures drawn from the United Nations' World Urbanization Prospects database [11]. Delineations of rurality versus urbanity are based on country-specific definitions.

The second core indicator is measured through a special survey periodically conducted by WHO among national health authorities [9]. The 2010 survey round obtained 32 updated reports from Countdown countries, representing half (47%) of them. The survey included new questions on HRH planning and competency frameworks. We analyse competencies and authorization to perform emergency obstetric and neonatal care signal functions [12,13] among different categories of providers, as a proxy for the capacity of health systems to efficiently use the human resources already available. We also monitor existence of policies authorizing community-based health workers to identify and treat pneumonia, in line with international recommendations on community based management of sick children [14].

Lastly, we use the new survey data to assess coverage of strategic plans for health workforce management and development in the Countdown countries. The existence of a documented HRH plan may be considered a proxy indicator of technical and institutional capacity (governance and leadership) of ministries of health to implement HRH policies at national level for improved health outcomes [15].

## Results

### Health workforce density and situation in 68 low- and middle-income countries

**Figure 1:**
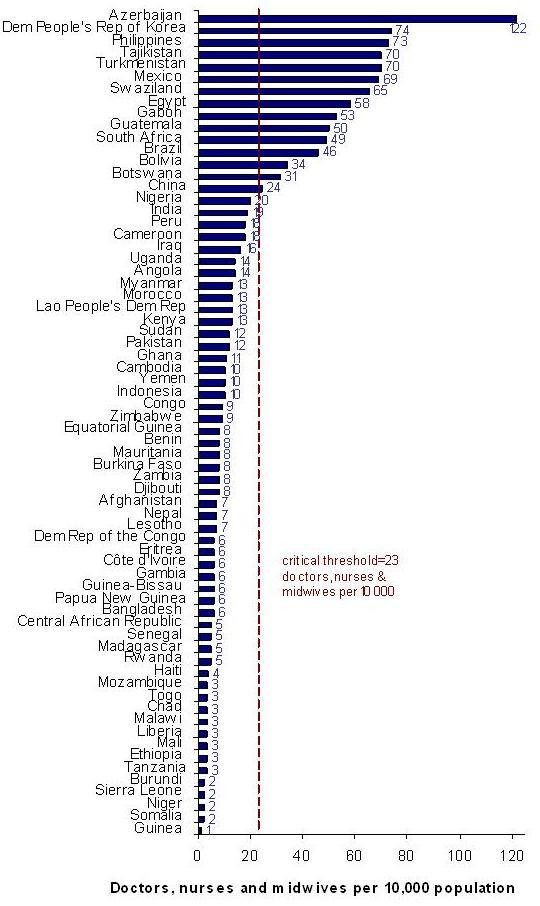
**Density of doctors, nurses and midwives in the 68 Countdown priority countries**. Source: WHO Global Atlas of the Health Workforce.

In the most recent estimates [10], 53 of the 68 priority countries have a national density of doctors, nurses and midwives that falls below the minimum threshold (23 per 10 000 population) established by the World Health Organization for countries to obtain adequate coverage rates for selected priority maternal, newborn and child health-care interventions [16] (Figure 1). This marks a marginal improvement compared to the situation reported in 2008, when 54 of the same set of countries had a workforce density below this threshold [9]. The median density across the 68 countries remained stable over the two-year period of observation at about 9 per 10 000 (results not shown). Most Countdown countries, especially in sub-Saharan African countries such as Burundi, Chad, Ethiopia, Guinea, Liberia, Malawi, Mali, Mozambique, Niger, Rwanda, Sierra Leone, Somalia, United Republic of Tanzania and Togo--and also elsewhere, e.g. Afghanistan, Bangladesh, Haiti, Nepal, Papua New Guinea--continue to experience critical shortages of skilled health personnel (see Figure 1).

Some countries showed improvements in workforce supply (including the Burkina Faso, Egypt, Mexico, the Philippines and Uganda), but only China moved above the threshold: from 21 per 10 000 reported in 2008 to 24 reported two years later. This trend may be partly related to national efforts to develop their health workforce: in 2002, the Ministry of Health implemented policies for improving medical and nursing education and increasing the numbers of health workers to support implementation of the country's HRH strategic plan [17]. However, such apparent changes in workforce density may result from inconsistencies in classification and measurement, particularly of doctors, who outnumber nurses. It is possible that official statistics on doctors may be underestimated or overestimated, especially in the context of a rapidly growing private health sector and with the inclusion of clinical practitioners without advanced medical training, who constitute a sizeable proportion of the Chinese health workforce [18,19].

Innovative strategies have been implemented in many Countdown countries to rapidly scale up the health workforce, especially in the context of primary health care renewal. For instance the Nigerian national government has allocated funds for the establishment of its Midwives Service Scheme, an initiative conceived as a collaborative effort across three tiers of government supported by strategic partners for mobilizing midwives in the delivery of essential MNCH services [20]. Under the scheme, midwives are training in life-saving skills and integrated management of neonatal and childhood illnesses, and deployed to rural areas where they receive continuous support from community based development committees. As of mid-2010, some 2500 newly qualified, previously unemployed and retired midwives had been deployed to 652 primary health care facilities. There is general consensus among stakeholders that the scheme has catalyzed renewed efforts in maternal mortality reduction and reports indicate increases in MNCH service utilization in target areas.

**Figure 2:**
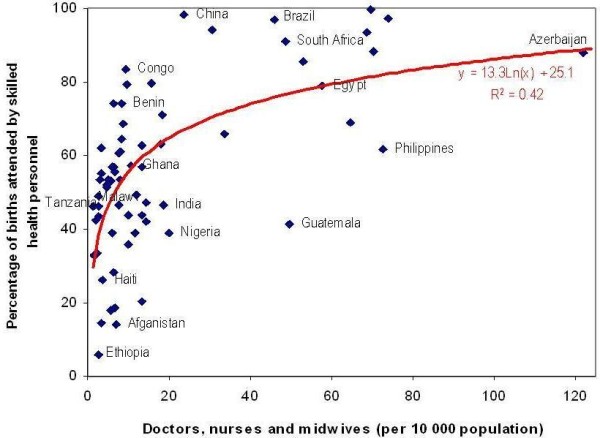
**Density of doctors, nurses and midwives versus coverage of skilled birth attendance, 68 countries**.

Overall, as expected, greater national supply of doctors, nurses and midwives is found to be strongly and positively correlated with improved coverage of deliveries by skilled health personnel across the 68 Countdown countries (correlation coefficient of 0.42) (see Figure 2). Women's access to skilled care during pregnancy and childbirth to ensure prevention, detection and management of complications is key to reducing maternal and neonatal mortality, and is one of the core MDG indicators.

However better evaluation is needed of the impacts of HRH supply on MNCH outcomes. Some countries still struggle to achieve high coverage rates of skilled birth attendance despite having relatively greater numbers of trained personnel: supply alone is not necessarily the main limitation to improved MNCH outcomes. In particular, some of the newly independent states of the former Soviet Union (Azerbaijan, Tajikistan, Turkmenistan) inherited workforces that were designed to provide health care accessible to all, with high staffing norms, but are now considered ill-suited to the demands facing modern health care systems. One of the greatest challenges for ministries of health in these contexts is to keep huge bodies of staff up-to-date with new developments. However, often in-service training has been minimal, post-independence, and many trained personnel have left the health sector or even the country altogether (but may still be tallied in workforce statistics) [21,22].

Furthermore, national averages of workforce density often hide marked inequalities in distribution, such as across geographical areas (e.g. urban/rural) and employment sectors (public/private). South Africa is a case in point. While the country's overall density of doctors and nurses is above the previously mentioned threshold, only 31% of registered medical practitioners and 59% of nursing personnel work in the public sector [23]. A large majority of medical specialists work only in the private sector. Yet barely 20% of the population accesses private health services. Some of these data may be overestimated: counts of doctors and nurses in public service are derived from the personnel salary administrative system, but the total number registered may include many who are not working at all due to unemployment, illness or other reasons. For instance workplace absences due to illness are likely increasing over time as a result of the high prevalence of HIV/AIDS; a national survey done in 2002 found a 16% HIV prevalence rate among health workers [24]. Meanwhile vacancies in the public sector remain high: 35% of medical practitioner positions and 40% of professional nurse positions stood vacant in 2008 [23].

**Figure 3:**
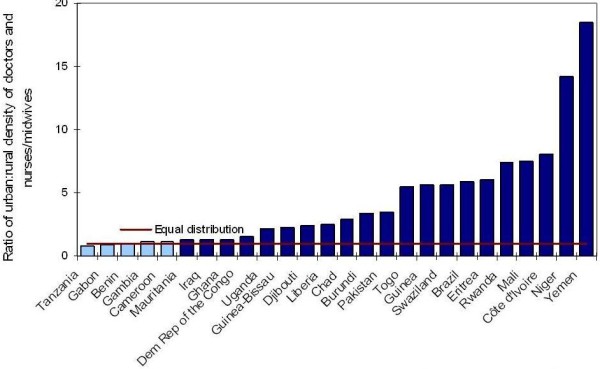
**Urban: rural distribution of doctors and nurses/midwives in 26 countries**. Source: WHO Global Atlas on the Health Workforce and authors' calculations.

A crucial challenge to many countries like South Africa, more than simply workforce numbers, is their distribution and functioning, with marked imbalances across sectors and locations. As seen in Figure 3, of those countries with available data, only a handful (Benin, Cameroon, Gabon, the Gambia and the United Republic of Tanzania) show equitable geographical distribution of doctors, nurses and midwives across urban and rural areas. The overwhelming majority of countries (81%) show a population-adjusted workforce strongly favouring urban areas. This can be related to many factors, including greater possibilities of private practice, relative unattractiveness of rural and remote areas due to poor working conditions (e.g. poor facilities, lack of supplies, including personal protective equipment), inadequate housing, limited opportunities for professional development, and limited educational opportunities for children.

In Brazil, for example, urban health professionals outnumber their rural counterparts six-fold (see Figure 3). To address disparities (i.e. inequity) in health outcomes, the Ministry of Health launched an initiative to reduce infant mortality in the country's poorer, more rural Northeast and Amazon regions [25]; it focuses on 256 municipalities that account for 50% of infant and neonatal deaths in these two regions of the country. Infant survival being closely linked to antenatal, delivery and postnatal care, the initiative also targets maternal health and survival. Action plans prioritize scaling up of family health teams, based on the Brazilian primary health care model [26], including expansion of production and deployment of nurses, obstetric nurses, nursing auxiliaries and community health workers. They also include the training of doctors and nurses in obstetric and neonatal urgencies and emergencies, and the recruitment of ambulance service providers (including doctors, nurses, emergency medical technicians and other support personnel) to ensure emergency care during transportation of pregnant women and newborns through the Mobile Emergency Attendance Service (Serviço de Atendimento Móvel de Urgência). Partnerships have been developed with universities and training centres to extend distance and online continuing education and learning programmes to support health service providers located in rural and remote areas. Other actions to improve workforce performance and retention include strengthening management capacities in the context of a decentralized health system, and effective regulatory and supportive frameworks such as recognition of community health workers by federal law and increasing their access to social security benefits.

### Who does what? Provider categories of MNCH services

The capacity of health systems to make efficient use of available human resources can be gauged, at least somewhat, through policies regarding skill mix and task sharing to supplement services. The roles of different categories of health workers were examined in relation to the regulation of provision of selected priority MNCH interventions along the continuum of care. According to WHO 2010 survey results, only 26 (38%) of the 68 Countdown countries had a policy allowing midwives to administer a set of lifesaving interventions during childbirth. This was essentially the same level as tallied two years earlier [8,9]. The interventions include administration of parenteral antibiotics, oxytocics and anticonvulsants; manual removal of placenta; removal of retained products of placenta; assisted vaginal delivery; and newborn resuscitation [12,13].

We further investigated the roles of specific categories of health workers (doctors, nurses, midwives and other practitioners) in relation to the regulation of provision of the signal functions, including also performing caesarean sections. As seen in Table 1, as expected, almost all Countdown countries authorized medical doctors to independently perform the full range of signal functions. Authorization for nursing and midwifery personnel is much less common. For example, in 2010 only two-thirds of the surveyed countries authorized nursing and midwifery professionals to perform manual removal of placenta; newborn resuscitation was authorized in about one in three countries. Only two countries with available data, the Gambia and Togo, authorized nurse-midwives to perform caesarean sections.

**Table 1:** Who is independently performing the signal functions for basic and comprehensive emergency obstetric and neonatal care in the Countdown countries?

	PERCENT OF COUNTRIES					
	Doctors	Midwives	Nurse-midwives	Nurses	Others	Doctors only
Administer injection magnesium sulphate for severe preeclampsia and eclampsia	100%	77%	90%	75%	57%	3%
Administer oxytocin for prevention of postpartum haemorrhage	100%	77%	94%	76%	57%	3%
Administer injectable antibiotics for sepsis in mother	100%	77%	94%	86%	62%	3%
Perform manual removal of placenta	100%	69%	63%	31%	55%	20%
Perform manual vacuum aspiration of products of conception	100%	52%	53%	32%	57%	30%
Prescribe oxytocin for induction/augmentation of labour	97%	52%	46%	22%	44%	30%
Ventilation of depressed newborn with self-inflating bag and mask	100%	33%	29%	11%	52%	37%
Perform Caesarean section	100%	0%	7%	0%	48%	50%

Source: WHO data 2010 (*N *= 32 Countdown countries).

On the other hand, many countries authorized other categories of clinical practitioners to perform the signal functions. About half of the surveyed countries had policies in place authorizing paramedical practitioners (aside from medical doctors and nursing or midwifery professionals) to perform each of the signal functions. Such findings underline important differences across countries in health worker training requirements, regulations and nomenclature. For instance, in Ethiopia health officers with three years of pre-service education in medicine and obstetrics and at least one year of internship following secondary school are authorized to perform caesarean sections, whereas in Liberia physician assistants with similar duration of training are not [27]. Many countries continued to retain a medical monopoly over essential clinical interventions, notably Mexico, where doctors alone were authorized to perform all of the signal functions.

In the area of child health, nearly half (29, or 46%) of the countries had a policy allowing community-based service providers (community health workers or other trained providers) to manage pneumonia in 2010, an important and rapid increase compared with the 2008 finding of one-quarter (18, or 26%) of countries with such policy in place [8,9]. For instance, in India the government has partnered with non-governmental organizations and WHO to provide basic training for community health workers in management of sick children [28]. In Malawi, community-based health surveillance assistants have been widely deployed as part of a nation-wide programme to facilitate access to and utilization of essential child health care services, especially in hard-to-reach areas.

### Strategic planning for HRH development in the Countdown countries

**Figure 4:**
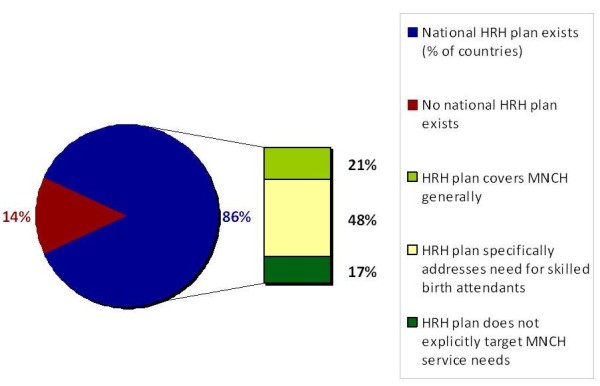
**Human resources planning for maternal, newborn and child health in Countdown priority countries**. Source: WHO data 2010 (N = 32 Countdown countries).

Effective management and development of human resources in health systems require top-level direction, informed by problems, solutions and evidence relevant to on-the-ground reality. A documented plan is one element of such direction. Based on available data, 86% of the Countdown countries have a national HRH management or development plan in place (see Figure 4). Most cover workforce planning for MNCH services, however only half (48%) of surveyed Countdown countries have an HRH plan that specifically addresses the need for skilled birth attendants based on national maternal and newborn health targets. Illustrative among those that do, the HRH plan for Lesotho includes explicit reference to strategic redeployment of specialist nurses to maternity and obstetric services at the hospital level based on the volume of maternity care demanded (drawing on a workload and task analysis), as well as health system requirements for medical specialists in obstetrics and gynaecology [29]. Zambia's plan targets and costs the scaling up of production of sufficient quantities of midwives as critical to improve maternal mortality rates [30].

In Malawi, efforts to improve the availability and accessibility of skilled health care providers, in order to impact maternal and child health, have been documented in the government's human resources strategy launched in 2004 [31]. The plan focuses on expanding domestic pre-service training capacity and outputs and improving retention (through salary top-ups, promotion opportunities and other incentives) of doctors, nurses, clinical officers and other priority cadres to raise personnel numbers to a level sufficient to deliver an essential health package. It also addresses using international consultants and volunteers as a stop gap, and bolstering planning, management and monitoring to identify short-term policy actions needed for the Ministry of Health to achieve medium-term HRH objectives. Indications of positive change have been reported: in 2007 there were 40% more doctors, 30% more nurses and 50% more clinical officers in post than in 2003 [32].

In Ghana, the challenge of providing equitable health services with inadequate numbers of skilled health workers has informed a strategy of expanding primary health care and close-to-client services following a series of national consultations on HRH that took place between 2003 and 2006. This strategy focused on the production of certain cadres, including midwives, community health officers with midwifery skills, primary heath care technical officers, health extension workers and medical assistants [33]. The strategy took into account the cost effectiveness of producing and retaining workers, especially in rural areas. To ensure rapid scale up of access to health workers, each of the country's ten regions was tasked to set up a community-oriented training school. Access to midwives was improved by increasing the numbers of new trainees through revision of midwifery training, from the former two-year post-basic training program to a straight three-year program for senior high school graduates. Increased intake in medical assistant training programs led to increases in the numbers of medical assistants at rural health centres providing care for newborns and children. Intake was simultaneously increased in medical and nursing education facilities in order to produce more highly skilled professionals to ensure referral support and supervision for other categories of staff. The data and evidence used to inform the planning process for the interventions were the geographical distribution of health workers by category of staff, and population age distribution in the country. The training of medical assistants was stepped up once it was realized from demographic analysis of HRH data that more than half of the practicing medical assistants and midwives were due for retirement.

## Discussion

We tracked a series of indicators and reviewed case studies for better understanding human resources for maternal, newborn and child health in 68 low- and middle-income countries prioritized for action by the Countdown to 2015 Initiative. Slow progress in HRH remains one of the most serious challenges for health systems across these countries. Most (78%) of the 68 countries face acute shortages of doctors, nurses and midwives. Traditional solutions for scaling up numbers of highly skilled personnel are unlikely to yield significant improvements in the short term, given the lengthy periods required to see the effects of training efforts (e.g. up to eight years in the case of educating new doctors). Moreover large variations are observed within and across countries. In many cases, workforce maldistribution across areas and sectors represents a larger challenge than absolute numbers for health systems to reach underserved populations. This paper has highlighted progress and lessons learnt from countries in adapting to HRH challenges through evidence-informed decision making.

Many Countdown countries are investing in comprehensive strategies to achieve a sufficient and equitably distributed health workforce to meet health systems goals. We found a strong and positive correlation between availability of doctors, nurses and midwives in countries and coverage of attendance during childbirth by a skilled provider, the latter being one of the core indicators for monitoring progress towards the MDGs. Key priorities for HRH development include: rapidly increasing the outputs of health professions education programmes in countries with critical shortage; measures to improve supervision, technical capacity and performance of health workers; actions to enhance worker retention, including in rural and underserved areas; and addressing workforce imbalances in terms of distribution, skills mix and skills utilization. Task sharing (e.g. allowing more cadres to perform signal functions for emergency obstetric and neonatal care or manage common childhood illnesses), strengthening policy effectiveness and establishing national HRH strategic plans based on solid data are all good signs of progress. However survey data confirm that many countries continue to retain a medical monopoly over essential clinical functions, despite having inadequate numbers and inequitable distribution of doctors. At the same time, findings presented here from a survey of health ministries pointed to a greater need to synergize systems-wide HRH planning with priority service delivery areas, notably MNCH services.

The need remains for more systematic, reliable and comprehensive data and information on HRH at the national and global levels to support planning, decision making and research. International calls are growing for improved collection, analysis and translation of information into evidence that can be used for HRH policy, planning, programming and accountability [16,34-36]. This analysis was limited by partial data availability and by heterogeneity in the information sources accessed. For example, workforce density data collated in the WHO's Global Atlas are dependent on the nature of the original source; it is not always certain how well national statistics capture (or not) private sector employment, workforce attrition and other labour market dynamics [10]. Imprecise professional boundaries and differences in defining and categorizing certain types of health workers present ongoing challenges in capturing and analyzing health workforce data within and across countries and over time [37].

Our findings highlight that nurses, midwives, community health workers and other service providers are often characterized in different settings by different training requirements, scopes of work and practice regulations. In order to monitor trends in health workforce situation and performance, or for countries to share experiences and best practices, it is necessary to know how health workers are defined and classified in the original information sources. We recommend that future efforts in measuring and monitoring human resources for MNCH adopt international standard classifications for social and economic statistics (or their national equivalents), including those relevant to the health workforce. In particular, the latest revision to the International Standard Classification of Occupations (known as ISCO-08) offers a universal system for classifying and aggregating occupational information across national economies according to assumed differences in skill level and skill specialization, and can serve as a model to facilitate communication about health occupations, regardless of variations in training requirements, regulations and nomenclature [38]. The tool may not capture the full complexity and dynamics of national health labour markets, but it can be useful for mapping different categories of human resources for purposes of statistical description and analysis, including those identified as critical to provision of MNCH services (Table 2). Notably, although paramedical practitioners and community health workers were not counted in workforce density figures measured here, improved reporting modalities in countries should lead to strengthening the global information and evidence base on these cadres over time. However, measuring appropriately the situation in contexts of large numbers of disparate cadres raises more questions. For instance, given differences in scopes of work and levels of care provided, should some form of weighting be used in calculating workforce-population ratios to account for such differences [39]? Moreover, density figures alone do not necessarily take into account all of a health system's objectives, particularly with regard to accessibility, equity, quality and efficiency.

**Table 2:** Classifying health workers: main categories of human resources for maternal, newborn and child health in the International Standard Classification of Occupations (2008 revision)

Occupational title	ISCO code*	Definition
**Health services managers**	1342	Plan, direct, coordinate and evaluate the provision of clinical and community health care services, e.g. health facility administrator, clinical director, community health care coordinator
**Generalist medical doctors**	2211	Study, diagnose, treat and prevent illness, disease, injury and other physical and mental impairments and maintain general health in humans through application of the principles and procedures of modern medicine, e.g. general practitioner, family medical practitioner, primary care physician
**Specialist medical doctors**	2212	Study, diagnose, treat and prevent illness, disease, injury and other physical and mental impairments using specialized testing, diagnostic, medical, surgical, physical and psychiatric techniques, e.g. obstetrician, gynaecologist, paediatrician
**Nursing professionals**	2221	Plan, manage, provide and evaluate nursing care services, e.g. clinical nurse, nurse practitioner, paediatric nurse, public health nurse
**Midwifery professionals**	2222	Plan, manage, provide and evaluate midwifery care services
**Paramedical practitioners**	2240	Provide diagnostic, curative and preventive medical services using advanced clinical procedures, e.g. clinical officer, surgical technician
**Nursing associate professionals**	3221	Provide basic nursing and personal care and health advice as per established care, treatment and referral plans, e.g. assistant nurse, enrolled nurse, practical nurse
**Midwifery associate professionals**	3222	Provide basic health care and advice before, during and after pregnancy and childbirth, e.g. assistant midwife
**Community health workers**	3253	Provide basic health education, preventive health care and home visiting services, e.g. community health aide, family health worker
**Medical assistants**	3256	Perform basic clinical and administrative tasks to support patient care under the direct supervision of a medical practitioner or other health professional

Source: Adapted from International Labour Organization [38].

* Note: Refers to the ISCO-08 code at the most disaggregated four-digit (unit group) level.

Planning, scaling up and monitoring of production, deployment and retention of human resources for MNCH involves a large number of stakeholders both inside and outside the health sector, including the ministry of health and local health authorities, as well as many others such as ministries of education, labour and finance, central statistics agencies, public service commissions, non-governmental organizations, health professional regulatory councils and associations, community councils and associations, and development partners. Effective strategies must respond to both the needs of the population and the expectations of health workers [40]. Solutions to HRH challenges require effective dialogue and partnership, including intersectoral approaches and interprofessional collaboration to address the necessary education, regulation, financing, and professional and personal support for health workers to improve access to and quality of comprehensive MNCH services. Countries and partners, such as the Countdown to 2015, should be encouraged and supported to monitor HRH development and its impacts on progress towards MDGs 4 and 5, identify knowledge gaps, and advocate for solutions supported by evidence to make a difference in the lives of women and children.

## Competing interests

The authors declare that they have no competing interests.

## Authors' contributions

NG and BM conceptualised the study design. NG prepared the first draft of the manuscript. BM and BD contributed to writing and interpretation of findings. AF, FN, MP, DS and HB contributed country case studies. All authors read and approved the final version.
